# Retrospective Analysis of Nodal Spread Patterns According to Tumor Location in Pathological N2 Non-small Cell Lung Cancer

**DOI:** 10.1007/s00268-012-1743-5

**Published:** 2012-09-05

**Authors:** Yoshihisa Shimada, Hisashi Saji, Masatoshi Kakihana, Hidetoshi Honda, Jitsuo Usuda, Naohiro Kajiwara, Tatsuo Ohira, Norihiko Ikeda

**Affiliations:** First Department of Surgery, Tokyo Medical University Hospital, 6-7-1 Nishishinjuku, Shinjuku-ku, Tokyo, 160-0023 Japan

## Abstract

**Background:**

The purpose of the present study was to determine the nodal spread patterns of pN2 non-small cell lung cancer (NSCLC) according to tumor location, and to attempt to evaluate the possible indications of selective lymph node dissection (SLND).

**Methods:**

We retrospectively analyzed nodal spread patterns in 207 patients with NSCLC of less than 5 cm with N2 involvement.

**Results:**

The tumor location was right upper lobe (RUL) in 79, middle lobe in 12, right lower lobe (RLL) in 40, left upper division (LUD) in 41, lingular division in 11, and left lower lobe (LLL) in 24. Both RUL and LUD tumors showed a higher incidence of upper mediastinal (UM) involvement (96 and 100 %, respectively) and a lower incidence of subcarinal involvement (15 and 10 %, respectively) than lower lobe tumors (UM; RLL 60 %, LLL 42 %; subcarinal: RLL 60 %, LLL 46 %, respectively). Among the patients with 24 right UM-positive RLL and 10 left UM-positive LLL tumors, 2 showed negative hilar, subcarinal, and lower mediastinal involvement, and cT1, suggesting that UM dissection may be unnecessary in lower lobe tumors with no metastasis to hilar, subcarinal, and lower mediastinal nodes on frozen sections according to the preoperative T status. Among the patients with 12 subcarinal-positive RUL and 4 subcarinal-positive LUD tumors, one showed negative hilar or UM involvement, suggesting that subcarinal dissection may be unnecessary in RUL or LUD tumors with no metastasis to hilar and UM nodes on frozen sections.

**Conclusions:**

The present study appears to provide one of the supportive results regarding the treatment strategies for tumor location-specific SLND.

## Introduction

Lobectomy with systematic mediastinal lymph node dissection (LND) has been considered the standard of care for resectable non-small cell lung cancer (NSCLC). Lymph node dissection was first reported by Cahan in 1960 [1] and is known to enhance staging accuracy by increasing lymph node harvesting and improving the identification of occult N2 disease. In contrast, other investigators claim that LND can potentially increased postoperative morbidity or may require longer operative time [2–5]. Some randomized controlled trials addressing the survival benefit of LND and mediastinal lymph node sampling showed no difference in survival outcome between patients undergoing LND and those undergoing lymph node sampling [3, 6, 7]. Whether or not patient outcome is improved by LND remains controversial.

At present, early lung cancers are more frequently encountered because of the widespread use of high-resolution computed tomography (CT) in routine practice and cancer screening [8, 9]. Therefore, the extent of LND should be tailored to each patient. Selective lymph node dissection (SLND) based on the tumor location-specific lymphatic pathway should be undertaken especially for patients with no apparent lymph node metastasis or with impaired pulmonary function, or for elderly patients. In the present study, we retrospectively reviewed the prevalence of lymph node involvement in each mediastinal region in patients with N2 NSCLC according to the location of the primary tumor, and we attempted to evaluate the possible indications for SLND.

## Patients and methods

### Patients

From January 1990 to December 2007, a total of 2,195 patients underwent radical surgical resection of at least a lobectomy and systematic LND for NSCLC at our hospital. Of these 2,195 patients, we retrospectively analyzed lymph node spread patterns and outcome in 207 patients with NSCLC of less than 5 cm with N2 involvement. We excluded patients who had received preoperative treatment, including chemotherapy or chemoradiotherapy, those who had undergone only biopsy and SLND, and those who had low-grade malignant tumors. We also excluded patients with tumors spreading across lobar fissures and invading multiple lobes.

Preoperative evaluation included physical examination, chest radiography, computed tomography (CT) of the chest and abdomen, magnetic resonance imaging of the brain, bone scintigraphy, and blood examination. We determined that a large lymph node over 10 mm in the shortest axis was positive for metastasis on CT scans. Positron-emission tomography (PET) scan (recently integrated PET-CT scan) was not routinely used for staging resectable tumors during the study period. In recent years, endobronchial ultrasound-guided transbronchial needle aspiration (EBUS-TBNA) was sometimes performed for the patients having suspected multiple N2 lymph node metastases, but it was not routinely used. Similarly, mediastinoscopic biopsy was not routinely performed. Patients with N2 lymph node positively diagnosed by EBUS-TBNA or mediastinoscopic biopsy were excluded from the group of operative indication candidates.

The stage of disease was determined according to the 2009 7th Edition of the TNM Classification for Lung and Pleural Tumors [10]. The institutional review board of our institution approved the data collection and analyses and waived the need to obtain written informed consent from each patient.

### Operation

During thoracotomy, lymph nodes in the ipsilateral thoracic cavity were completely resected. Systematic nodal dissection, including the superior to inferior mediastinum, was then performed after pulmonary resection. In cases of left thoracotomy, upper mediastinal dissection indicated aortic and tracheobronchial node dissection. If intraoperative findings showed that hilar or mediastinal lymph nodes were highly suspicious for metastatic disease, the resected lymph node specimens were immediately examined pathologically in frozen sections. Whether or not the presence or absence of lymph node metastasis was judged by intraoperative diagnosis, systematic LND was performed in the present study patients. Mediastinal metastases were considered to be skip metastases if any of the N2 nodes, but not the N1 nodes, were involved.

Mediastinal lymph node stations were grouped into the “zones” proposed by the International Association for the Study of Lung Cancer (IASLC) lung cancer staging project [11]. We also reviewed the correlation between nodal zone spread pattern and tumor location. We classified lymph node stations into the following six zones: the right upper (RU) and left upper (LU) zones, each including #2R, #3a, #3p, and #4R nodes; the subcarinal (SC) zone, including #7 nodes; the right lower (RL) and left lower (LL) zones, each including #8 and #9 nodes; and the aortic-pulmonary (AP) zone, including #5, and #6 nodes.

### Statistical analysis

Overall survival time was measured from the date of surgery to the date of death from any cause or the date on which the patient was last known to be alive. Survival curves were plotted according to the Kaplan–Meier method and compared with the log-rank test. Two-category comparison was performed by the Pearson χ^2^ test and Fisher’s exact test for quantitative data. All tests were two-sided, and *p* values of <0.05 were considered to indicate statistically significant differences. We used StatView 5.0 (SAS Institute Inc., Cary, NC) for the statistical analysis.

## Results

**Fig. 1:**
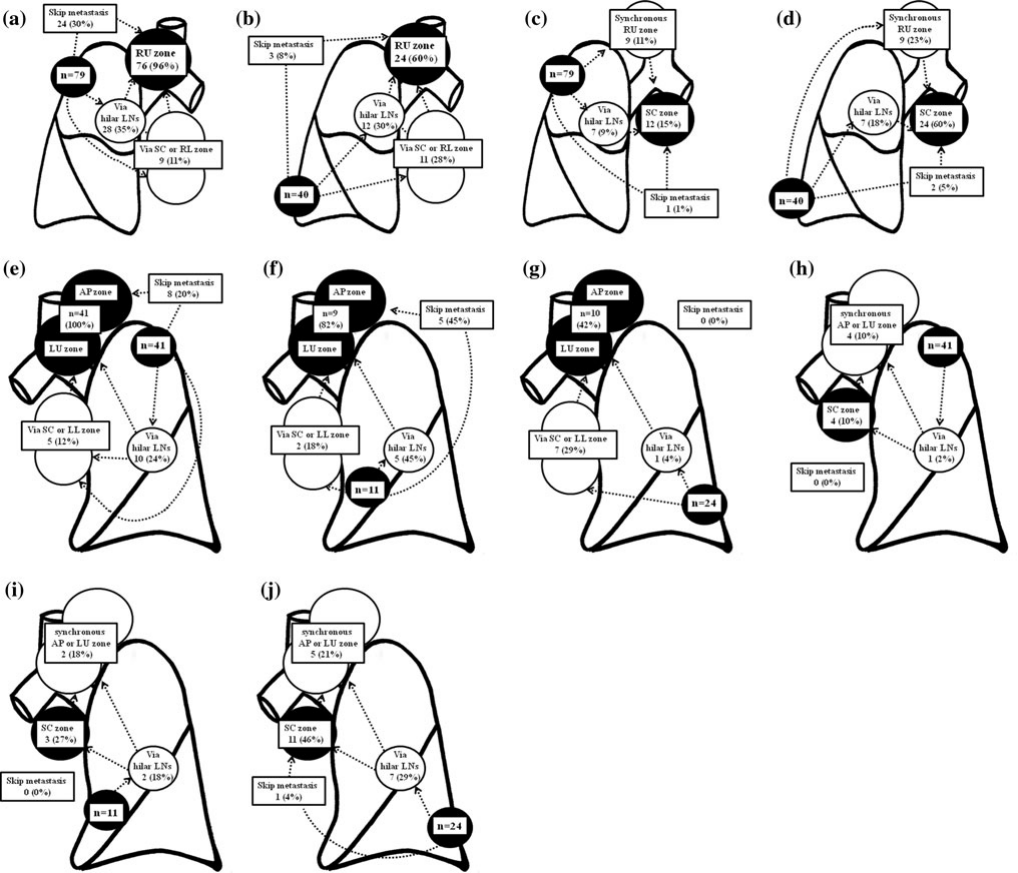
Lymph node spread patterns according to the primary tumor location: **a** tumors of the right upper lobe (RUL) and right upper mediastinal metastasis. **b** Tumors of the right lower lobe (RLL) and right upper mediastinal metastasis. **c** Tumors of RUL and subcarinal metastasis. **d** Tumors of RLL and subcarinal metastasis. **e** Tumors of the left upper division (LUD) and left upper mediastinal metastasis. **f** Tumors of the left lingular division (LLD) and left upper mediastinal metastasis. **g** Tumors of the left lower lobe (LLL) and left upper mediastinal metastasis. **h** Tumors of LUD and subcarinal metastasis. **i** Tumors of LLD and subcarinal metastasis. **j** Tumors of LLL and subcarinal metastasis

Patient characteristics are summarized in Table 1. Of the 207 patients with NSCLC of less than 5 cm with N2 involvement, 55 (27 %) had skip metastasis, and 97 (47 %) had both hilar and the remaining 55 patients had metastatic segmental lymph nodes or subsegmenta lymph nodes with mediastinal lymph nodes metastasis. In addition, 74 (36 %) were diagnosed with cN2 disease by the chest CT. Lymph node spread patterns according to primary tumor location are presented in Fig. 1. The most common site of involvement for tumors of the right upper lobe (RUL; *n* = 79) was the RU zone (*n* = 76; Fig. 1a). Right upper lobe tumors showed a significantly higher incidence of RU zone metastasis than right lower lobe (RLL) tumors (96 vs. 60 %, *p* < 0.001: Fig. 1a, b). In contrast, when RU zone metastasis was present, RLL tumors showed a significantly higher incidence of simultaneous metastasis to the SC or RL zone than RUL tumors (28 vs. 11 %, *p* = 0.026: Fig. 1a, b). The incidence of skip metastasis to only the RU zone was statistically lower among patients with RLL tumors than among those with RUL tumors (8 vs. 30 %, *p* = 0.005: Fig. 1a, b). Right upper lobe tumors showed a significantly lower incidence of SC zone metastasis than RLL tumors (15 vs. 60 %, *p* < 0.001: Fig. 1c, d). Most RUL tumors with SC zone metastasis showed simultaneous metastasis to the RU zone or hilar lymph nodes, and only one patient showed skip metastasis to the SC zone (Fig. 1c).

**Table 1:** Patient characteristics (*n* = 207)

	*n*	(%)
Overall	207	(100)
Sex		
Male	134	(65)
Female	73	(35)
Histologic type		
Adenocarcinoma	149	(72)
Squamous cell carcinoma	41	(20)
Others	17	(8)
Tumor size (cm)		
2.0	38	(18)
2.1–3.0	55	(27)
3.1–5.0	114	(55)
p-T status		
pT1	47	(23)
pT2	129	(62)
pT3	18	(9)
pT4	13	(6)
Hilar lymph node metastasis		
Present	97	(47)
Absent	110	(53)
Skip metastasis		
Present	55	(27)
Absent	152	(73)
Tumor location		
Right upper lobe	79	(38)
Right middle lobe	12	(6)
Right lower lobe	40	(19)
Left upper division	41	(20)
Left lingular division	11	(5)
Left lower lobe	24	(12)
Procedure		
Pneumonectomy	15	(7)
Bilobectomy	19	(9)
Lobectomy	173	(84)

The most common site of involvement for tumors of the left upper division (LUD) (*n* = 41) was the AP or LU zone (*n* = 41; 100 %: Fig. 1e). Left upper division tumors showed a significantly higher incidence of AP or LU zone metastasis than left lower lobe (LLL) tumors (100 vs. 42 %, *p* < 0.001: Fig. 1e, g). In contrast, when AP or LU zone metastasis was present, LLL tumors showed a higher incidence of simultaneous metastasis to the SC or LL zone than LUD tumors, but the difference was not significant (29 vs. 12 %, *p* = 0.089: Fig. 1e, g). The incidence of skip metastasis to only the AP or LU zone was 45 % in left lingular division tumors, 20 % in LUD tumors, and 0 % in LLL tumors, but the difference was not significant (Fig. 1e–g). Left upper division tumors showed a significantly lower incidence of SC zone metastasis than LLL tumors (10 vs. 46 %, *p* < 0.001: Fig. 1h, j). All LUD tumors with SC zone metastasis showed simultaneous metastasis to the AP or LU zone, but no patient showed skip metastasis to the SC zone (Fig. 1h).

**Fig. 2:**
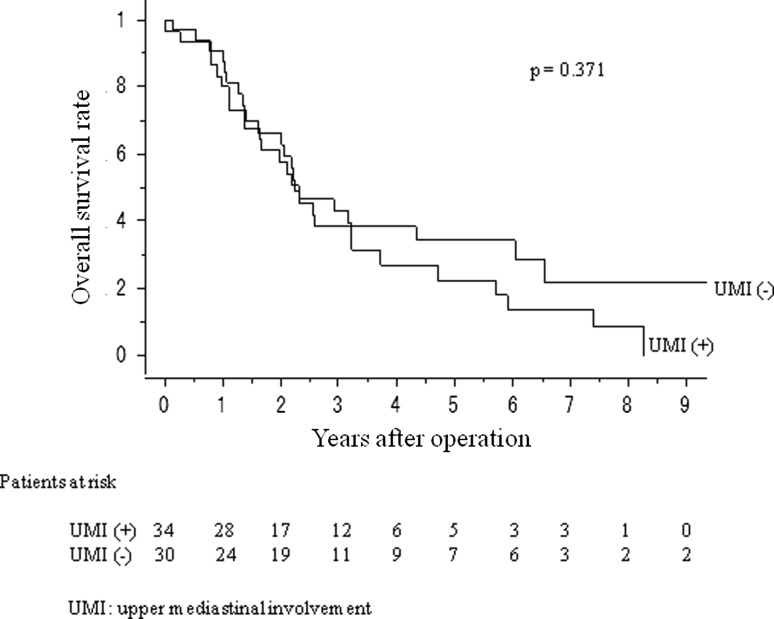
Overall survival curves of lower lobe non-small cell lung cancer (NSCLC) pN2 patients, with or without upper mediastinal metastasis

**Fig. 3:**
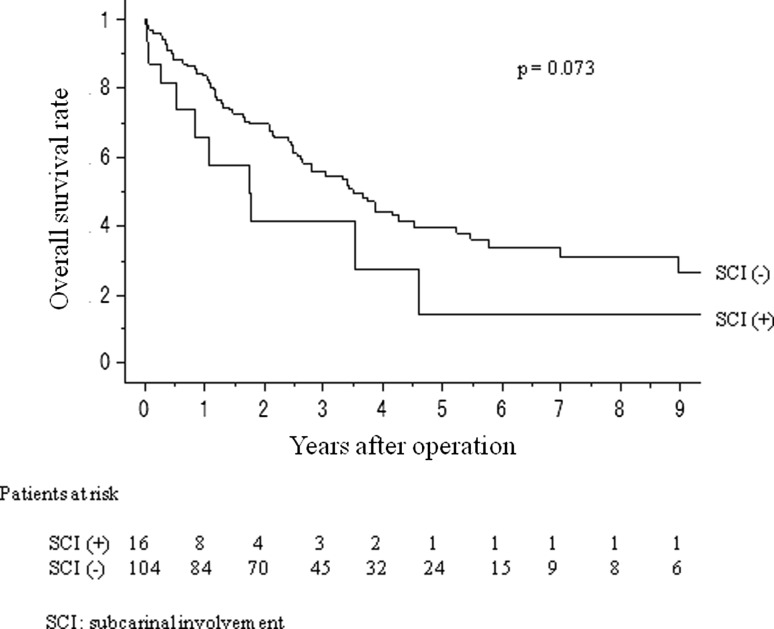
Overall survival curves of right upper lobe or left upper division NSCLC pN2 patients, with or without subcarinal metastasis

Patients were further categorized as those with tumors of the lower lobes (*n* = 64; 40 of right and 24 of left) and those with RUL or LUD tumors (*n* = 120; 79 of RUL and 41 of LUD). The prognosis of patients with lower lobe tumors and RUL or LUD tumors was analyzed. The 5-year overall survival (OS) rates of patients with tumors of the lower lobes with upper mediastinal metastasis (*n* = 34, 22 %) were poorer than, but not significantly different from, those of the patients without upper mediastinal metastasis (*n* = 30, 34 %) (*p* = 0.371; Fig. 2). The 5-year OS rates of patients with RUL or LUD tumors with SC zone metastasis (*n* = 16, 14 %) were poorer than, but not significantly different from, those of the patients without SC zone metastasis (*n* = 104, 40 %) (*p* = 0.073; Fig. 3).

**Fig. 4:**
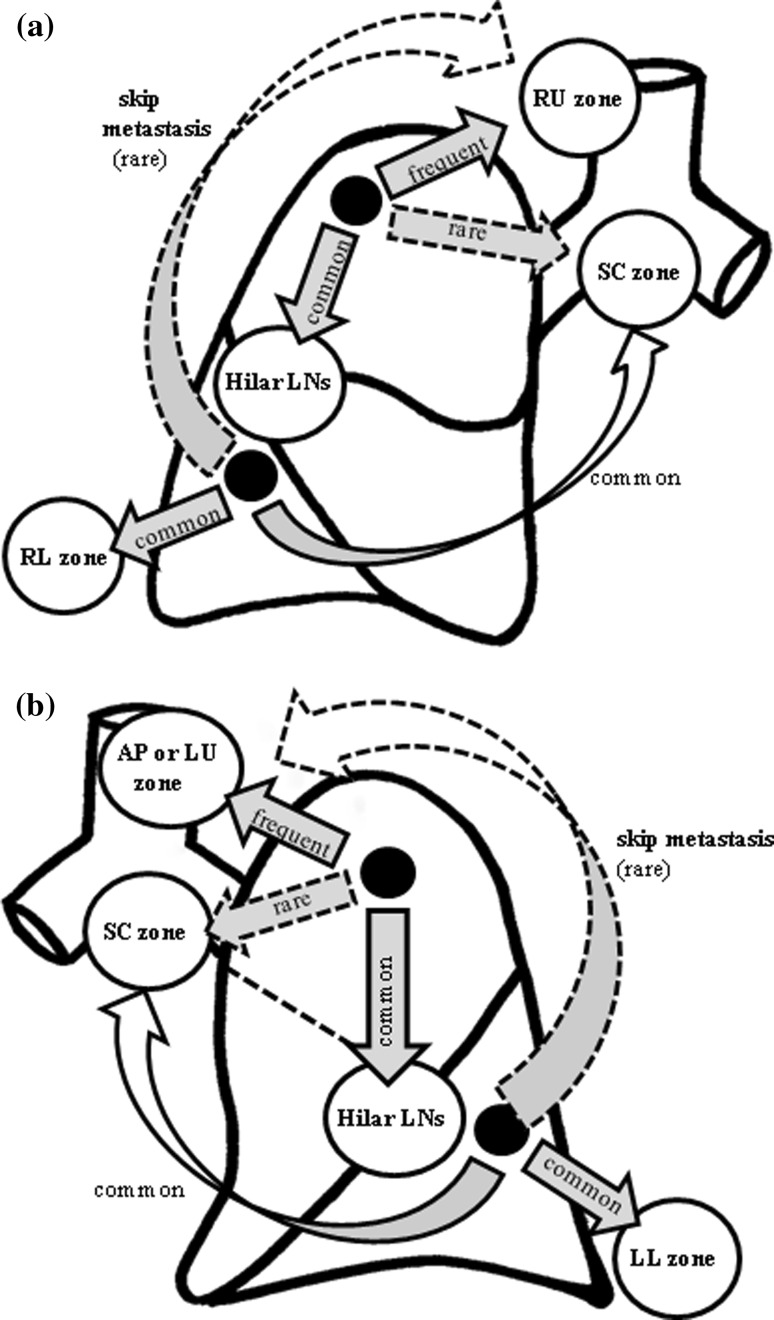
Diagrams of the main pathways of lymphatic spread according to tumor location. **a** In right-side tumors, almost all RUL tumors metastasized to the RU zone directly or through the hilar lymph node. RUL tumors metastasized less frequently to the SC zone.* Right lower lobe* (RLL) tumors metastasized to various mediastinal lymph node zones, and skip metastasis to the RU zone was rare in RLL tumors. **b** In left-side tumors, all LUD tumors metastasized to the AP zone directly or through the hilar lymph node.* Upper lobe* tumors metastasized less frequently to the SC zone.* Left lower lobe* (LLL) tumors metastasized to various mediastinal lymph node zones, and skip metastasis to the AP zone was rare in LLL tumors

The combined treatment strategies for tumor location-specific SLND in N2 NSCLC patients according to clinical T status are summarized in Table 2. Among 24 patients with upper mediastinal metastasis from RLL tumors, nine showed no evidence of hilar, SC zone, and lower mediastinal metastasis. Of these nine patients, only one had clinical T1. Similarly, among ten patients with upper mediastinal metastasis from LLL tumors, only one showed no evidence of hilar, SC zone, and lower mediastinal metastasis, and clinical T1 status. Upper mediastinal dissection may be unnecessary in lower lobe tumors with negative hilar, SC and lower mediastinal nodes on frozen sections if the preoperative T status is T1 (Table 3). In contrast, among 12 patients with SC zone metastasis from RUL tumors, one showed no evidence of hilar or RU zone metastasis, and that tumor was classified as clinical T2. Among four patients with SC zone metastasis from LUD tumors, none showed evidence of hilar, upper mediastinal metastasis. This finding supports the hypothesis that SC dissection may be unnecessary in RUL and LUD tumors with no metastasis to hilar and upper mediastinal nodes on frozen sections, regardless of the clinical T status. Figure 4 shows diagrams of the main pathways of lymphatic spread of tumors according to tumor location.

**Table 2:** Strategy for tumor location-specific selective nodal dissection in N2 non-small cell lung cancer (NSCLC) patients: distribution of upper mediastinal involvement according to clinical T status

Tumor location					
	RUL	RLL	LUD	LLD	LLL
	*n* (%)				
No. of patients with N2	79 (100)	40 (100)	41 (100)	11 (100)	24 (100)
No. of patients with UMI	76 (96)	24 (60)	41 (100)	9 (82)	10 (42)
Patients with UMI					
HI (–), SCI (–), LMI (–)	44 (56)	9 (23)	22 (54)	5 (45)	2 (8)
Clinical T1	14 (18)	1 (4)	5 (12)	2 (18)	1 (4)
Clinical T2–4	30 (38)	8 (21)	17 (41)	3 (27)	1 (4)

*RUL* right upper lobe, *RLL* right lower lobe, *LUD* left upper division, *LLD* left lingular division, *LLL* left lower lobe, *UMI* upper mediastinal involvement, *HI* hilar lymph node involvement, *SCI* subcarinal involvement, *LMI* lower mediastinal involvement

**Table 3:** Strategy for tumor location-specific selective nodal dissection in N2 NSCLC patients: distribution of subcarinal involvement according to clinical T status

Tumor location					
	RUL	RLL	LUD	LLD	LLL
	*n* (%)				
No. of patients with N2	79 (100)	40 (100)	41 (100)	11 (100)	24 (100)
No. of patients with SCI	12 (15)	24 (60)	4 (10)	3 (27)	11 (46)
Patients with SCI					
HI (–), UMI (–)	1 (1)	3 (8)	0 (0)	0 (0)	3 (13)
Clinical T1	0 (0)	1 (3)	0 (0)	0 (0)	0 (0)
Clinical T2–4	1 (1)	2 (5)	0 (0)	0 (0)	3 (13)

## Discussion

We set out to gain insight into the prevalence of lymph node metastasis in each mediastinal region in patients with pN2 NSCLC. The lymphatic pathways by which metastases from primary tumors in various segments and lobes spread toward the hilar and mediastinal lymph nodes have been investigated for over 50 years [12]. Studies of the patterns of location-specific lymphatic pathways of the lung have led to a better understanding of the importance of lymph node staging in the management of lung cancers. Although systematic LND consistently yields precise staging information, it may contradict the concept of the optimal extent of lymph node dissection based on the location of the tumor. Some authors have postulated that the dissection of lymph nodes without cancer cells causes higher morbidity and mortality because it extends the operative procedure [2, 6]. Moreover, the significance of LND regarding long-term outcome is still controversial. We therefore retrospectively reviewed the prevalence of mediastinal lymph node involvement in 207 patients with NSCLC of less than 5 cm with N2 involvement based on the location of the primary tumor, and we set out to determine the possible indications of location-specific SLND.

The IASLC staging project proposed the zone classification for future survival analyses [11]. Lee et al. [13] reported that grouping patients together according to zones provides accurate prognostic stratification for patients, and may resolve the ambiguity of the anatomical border, indicating applicability in the clinical setting. Therefore, we used the lymph node zone classification in this study.

Several retrospective studies have shown patterns of mediastinal lymph node metastases in relation to the location of the primary tumor [14–19]. Most of these studies have demonstrated that mediastinal lymph node metastases from RUL tumors occur predominantly in the RU area, but rarely in the SC area, whereas those from left upper lobe tumors occur most frequently in the AP or LU area, but those from tumors of the lower lobes rarely occur in the upper mediastinal area. In the present study, metastases to the SC zone from RUL or LUD tumors were significantly less frequent (15 and 12 %, respectively) than metastases to the SC zone from tumors of the lower lobes. The outcome of patients with RUL or LUD tumors with SC zone metastasis was poorer than, but not significantly different from, that of patients with RUL and LUD tumors without SC zone metastasis (*p* = 0.073). There was only 1 patient with only SC zone skip metastasis. Patients with upper lobe NSCLC involving SC nodes are reportedly rare [16, 18, 19], and they have poorer outcomes than those without SC node metastasis [19]. Based on these results, we also evaluated the possible indications of tumor location-specific SLND. Although we did not routinely perform frozen section diagnosis of sampled hilar lymph nodes, we conducted a frozen section examination intraoperatively if metastasis was suspected. There was only 1 patient with SC zone metastasis from RUL tumors who did not show any evidence of hilar and RU zone metastases, whereas no SC zone metastasis from any LUD tumors was observed when neither the hilar nor RU zone showed any evidence of metastasis. Resection of the SC zone in the case of RUL and LUD tumors may be unnecessary if neither upper mediastinal nor hilar lymph nodes show any evidence of metastasis on frozen sections, regardless of the clinical T status.

There were fewer patients with metastases to the upper mediastinal zone from tumors of the lower lobes than with metastases to the upper mediastinal zone from tumors of the upper lobes. The outcome of patients with tumors of the lower lobes with upper mediastinal metastasis was poorer than, but not significantly different from, that of patients with tumors of the lower lobes without upper mediastinal metastasis (*p* = 0.371). There was only one patient each with RU zone metastasis from a clinical T1 RLL tumor and AP zone metastasis from a clinical T1 LLL tumor, but neither showed any evidence of lymph node metastasis to the SC node, lower mediastinal zone, and hilum. Therefore resection of upper mediastinal zones in tumors of the lower lobes may be unnecessary even if the preoperative T status is T1, and if lymph node biopsies in the SC node, lower mediastinal zone, and hilum do not show any evidence of metastasis on frozen sections. However, former studies indicated that the superior and basal segment lung cancers in the lower lobe have different lymph node metastasis patterns [14]. Although there was no significant difference in the metastasis patterns of lower lobe tumors, this finding may be attributable to the small number of patients in the present study (data not shown). The strategy of lymph node dissection should be changed from extensive dissection to SLND, especially in early stage cancer or poor-risk patients, because SLND can reduce postoperative morbidity associated with such complications as bronchopleural fistula, chylothorax, or recurrent nerve palsy [2–5]. However, lung cancer can easily metastasize to the mediastinum, and therefore patient selection should be determined carefully. If patients are suspected of having advanced disease based on intraoperative findings, LND should be performed.

The present study has several limitations. It was a retrospective study, and possible bias may exist. First, we examined suspected hilar or mediastinal lymph nodes intraoperatively in frozen sections, but specific systemic sampling methodologies have been established and used in the past. Second, the number of patients in this study may be too small to draw any definitive conclusion. Third, current less-invasive staging modalities, including PET-CT or EBUS were infrequently used because of the inclusion of a large amount of data from old cases, collected at a time when these procedures were less well established. Thus we might have inadvertently performed some operations on undetected N3 disease.

In conclusion, we demonstrated the potential validity of refraining from resecting lymph nodes in the SC zone in cases of RUL or LUD tumors, or those in the upper mediastinal zone in the case of tumors of the lower lobes. Considering the fact that NSCLC patients can benefit from SLND, a prospective study is essential to confirm the effect of tumor location-specific SLND on survival and optimal postoperative treatment.
